# Replantation in Chronic Alveolar Abscess

**Published:** 1894-10

**Authors:** Geo. A. Sullivan

**Affiliations:** Albany, N. Y.


### REPLANTATION IN CHRONIC ALVEOLAR ABSCESS.

BY GEO. A. SULLIVAN, D.D.S., ALBANY, N. Y.

Conservative dentistry has made rapid progress in recent years, and the dental profession must look with favor upon an operation by which diseased teeth can be restored to health and usefulness after all ordinary methods of treatment have failed.

Every dentist is familiar with the distressing condition presented in cases of chronic alveolar abscess and acute and constant pericementitis, cases which, in spite of every effort, will continue in a more or less inflamed condition. My method of treatment in those cases may be of interest.

My experience has extended over a period of six years, and in that time I have extracted and replanted fifteen teeth, four of which were lower molars, and I cannot report a single failure.

The operation of extracting and replanting an anterior tooth is a very simple one, and, if done quickly, the tooth need not be out of the mouth longer than ninety seconds, and I may add that it is an unnecessary waste of time to cut off the end of the root and cap the apex with gold. If the tooth is a little longer than the adjacent ones, it is much better to trim off the cutting-edge. I have only found it necessary in cases of bicuspids. I believe most failures are caused by sacrificing too much pericementum.

In performing this operation, prepare a glass of boiled water, to which add three or four drops of five-per-cent. alcoholic solution of hydronaphtol; wet a corner of a napkin in this water, and when the tooth is extracted wrap the wet corner of the napkin around it, cut off the abscess sac,—but as little pericementum as possible,—and remove all septic matter from the pulp-canal by drilling from the apex towards the crown; moisten the pulp-canal with chloroform by using a broach with a few fibres of cotton wound around it; insert a gutta-percha canal-point, and trim off the protruding end even with the end of the root; syringe out the alveolar socket with the prepared antiseptic water, insert the tooth, and ligate it to the adjacent teeth.

In two weeks the tooth will be as firm as any in the mouth.

I cannot understand why a dentist will treat a case of chronic alveolar abscess from six months to two years when the tooth can, by this operation, be restored to health in two weeks.

